# The lateral septum mediates kinship behavior in the rat

**DOI:** 10.1038/s41467-020-16489-x

**Published:** 2020-06-22

**Authors:** Ann M. Clemens, Hong Wang, Michael Brecht

**Affiliations:** 1 Bernstein Center for Computational Neuroscience Berlin, Humboldt-Universität zu Berlin, Philippstr. 13, Haus 6, 10115 Berlin, Germany; 2 CAS Key Laboratory of Brain Connectome and Manipulation, The Brain Cognition and Brain Disease Institute (BCBDI), Chinese Academy of Sciences, Shenzhen-Hong Kong Institute of Brain Science-Shenzhen Fundamental Research Institutions, Shenzhen Institutes of Advanced Technology, Xueyuan Boulevard, Shenzhen, 518055 China

**Keywords:** Cellular neuroscience, Sensory processing, Social behaviour

## Abstract

Evolutionary theory and behavioral biology suggest that kinship is an organizing principle of social behavior. The neural mechanisms that mediate kinship behavior are, however, not known. Experiments confirm a sibling-approach preference in young rat pups and a sibling-avoidance-preference in older pups. Lesions of the lateral septum eliminate such kin preferences. In vivo juxta-cellular and whole-cell patch-clamp recordings in the lateral septum show multisensory neuronal responses to kin and non-kin stimuli. Non-kin odor-responsive neurons are located dorsally and kin-odor responsive neurons are located ventrally in the lateral septum. With development, the fraction of kin-responsive lateral septal neurons decrease and ongoing firing rates increase. Lesion effects, developmental changes and the ordered representation of response preferences according to kinship—an organization we refer to as nepotopy—point to a key role of the lateral septum in organizing mammalian kinship behavior.

## Abstract (web summary)

Kinship behavior in rodents has been documented in the laboratory setting but the neural mechanisms that mediate kinship behavior are not known. Here, the authors show that the lateral septum has a key role in organizing mammalian kinship behavior.

## Introduction

The theory of inclusive fitness, proposed by Hamilton, posits that the evolutionary success of a gene is enhanced with support for the survival and reproduction of one’s genetic relatives^1^. This concept was an early subject of discussion by Darwin^2^ and gained later support from the detailed analysis of eusocial insect species where complex societies and social behaviors are organized to support genetic relatives^3^. Remarkably, even plants^4^ and single-cellular organisms^5^ show kin recognition, suggesting that kinship might be a social behavior older than the brain. In humans, kinship behavior is reflected by the tendency to form and maintain close bonds within families. The occurrence of kin preferences outside of the private domain is perhaps even more remarkable. One of the most famous examples of such behavior is the practice of popes to elevate nephews and other relatives to the cardinalate. Since then, both clerical and public institutions have enacted laws prohibiting nepotism (from Latin: *nepos*, nephew) to ensure that the individual’s kin preferences do not annul achievement and qualification. Here, we investigate the neural origins of kin preferences in rats. Kinship behavior in rodents is documented in the laboratory setting^6–8^, however, little is known about the brain structures and neuronal circuits which represent kinship.

## Results

### Sibling preference behavior in the rat

**Fig. 1:**
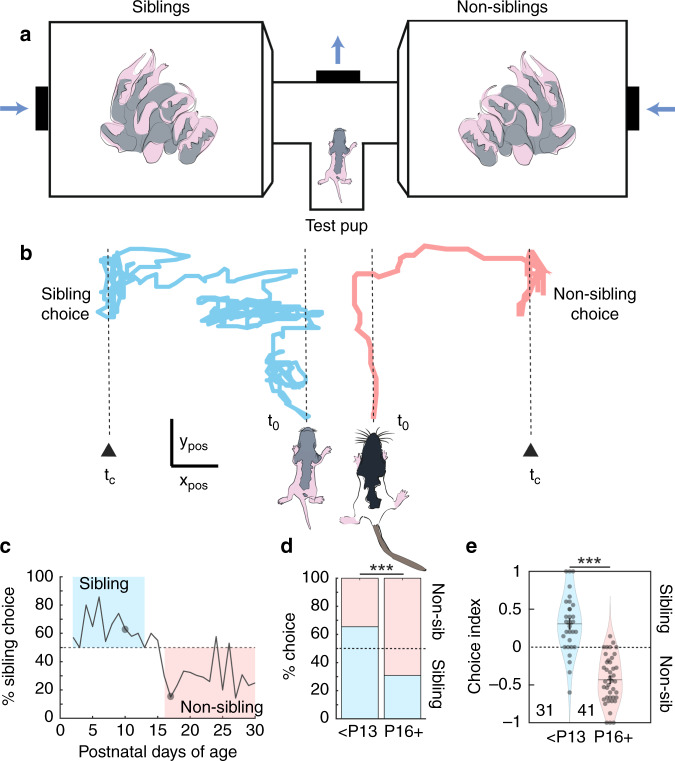
Development of kinship preferences in the rat. **a** Apparatus for sibling-preference testing. Test pup was placed in the T-tube with two opposing chambers containing sibling and non-sibling rats. Air was blown toward the center of the T-choice tube. **b** Young pups and older pups proceeded to the T-choice point and performed odor-sampling behavior before choosing a direction to crawl. Start of trial (t_0_) and end of trial with choice (t_c_). Young pups (left) crawled with prolonged latency, while older pups (right) proceeded to the choice point with shorter latencies and in a more direct route (scale: left pup, x–y pos: 2 cm; right pup, x–y pos: 3 cm). **c** Proportion of pups performing sibling and non-sibling choices with age. Pups were tested one to two times daily with trials separated by morning and afternoon. Gray dots indicate the time point of the example trajectories above. **d** Comparison of preference scores for sibling-preferring age group vs. non-sibling-preferring age group. Age divisions determined as previously indicated by Hepper^7^. Bars represent proportions of categorical choices pooled across animals (<P13: 140 sibling choices, 74 non-sibling choices; P16+: 112 sibling choices, 251 non-sibling choices; <P13 vs. P16+ choices: *p* = 6.2e−16, Fisher’s exact test). **e** Comparison of choice behavior on individual pup basis. 31 pups in the young pup group (<P13) vs. 41 pups in the older pup (P16+) group (*p* = 1.6e−13, unpaired *t*-test). Horizontal lines represent the mean, error bars are s.e.m. All tests are two-tailed. For detailed statistical information, see Supplementary Table 1. ***p < 0.001.

Kinship behavior in the rat was first demonstrated by Hepper, who showed that from birth, pups recognize and respond preferentially to genetic relatives^7,8^. Similar to Hepper, we built a two-chamber apparatus connected by a center T-tube where air was blown through each box to the center of the T where a third fan exhausted the odor (Fig. 1a). When rat pups were placed at the inlet of the T-tube, they showed odorant sampling behavior and then chose to crawl into either side of the T-tube (Fig. 1b). We tested rat pups one to two times per day and recorded a categorical preference score for each animal. A larger proportion of rats chose the sibling side compared with the non-sibling side until 13–15 days of age when the preference neared chance levels. From 16 days of age and on, the population of rats showed a preference for the non-sibling side (Fig. 1c). We compared behavioral choices at ages younger than P13 with the choices 16 days of age and older. In line with Hepper, we saw a clear divide of behavioral kin preference with age (Fig. 1d, e).

### Lateral septum lesions disrupt sibling preference in young pups

**Fig. 2:**
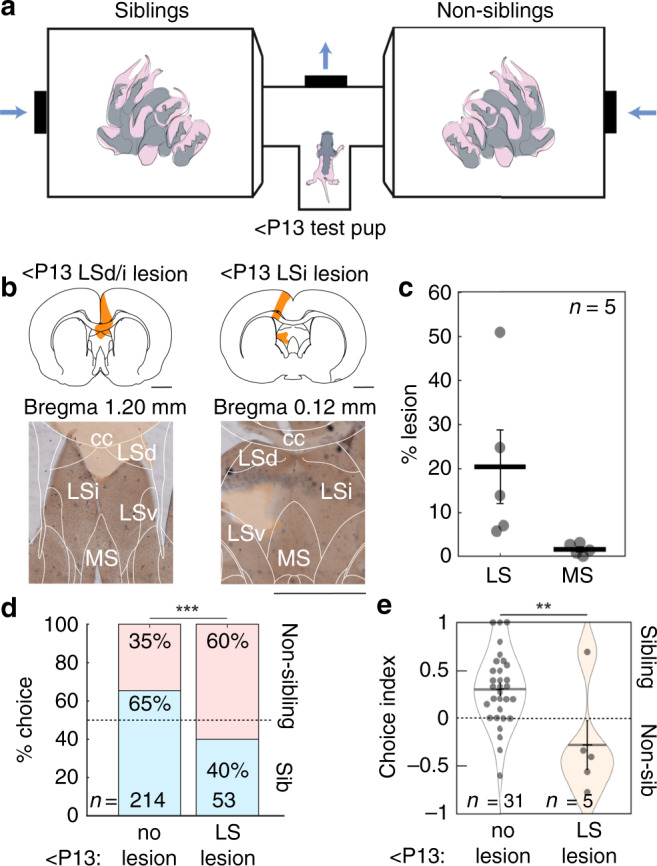
Sibling preference in young pups is disrupted by lesion of the lateral septum. **a** Apparatus for sibling-preference testing. Test pup was placed in the T-tube with two opposing chambers containing sibling and non-sibling rats. Air was blown toward the center of the T-choice tube. **b** Examples of two sections from two pups with lateral septal lesions. Left, lesion of dorsal and intermediate lateral septum (LSd/i) with overlying cortex. Right, lesion of intermediate lateral septum (LSi) and cortex (lesion area in orange). Scale bar applies to left and right micrographs. **c** Sections (100 µm thickness) of the five lesioned brains were analyzed for lesion extent. Average percent lesion of septum was estimated (LS: 20.45 ± 8.36%; MS: 1.53 ± 0.56%). Data represent the mean ± s.e.m. **d** Sibling preference in intact animals and with lateral septum lesion. Bars represent proportions of categorical choices pooled across animals (<P13 No lesion (as in Fig. 1d): 140 sibling choices, 74 non-sibling choices; <P13 lateral septum lesion: 21 sibling choices, 32 non-sibling choices; *p* = 8.9e−4, Fisher’s exact test). **e** Sibling choice behavior by animal, non-lesioned data as in Fig. 1e (<P13 No lesion vs. LS lesion: *p* = 0.005, *t*-test). Data represent the mean ± s.e.m. All tests are two-tailed. For detailed statistical information, see Supplementary Table 1. LSd dorsal lateral septum, LSi intermediate lateral septum, LSv ventral lateral septum, cc corpus callosum, MS medial septum. Scale bars: 2 mm. **p < 0.01, ***p < 0.001.

We next asked which brain structures may be responsible for the observed kinship behavior. We chose the lateral septum as a target, which is recognized for its role in social behavior, social memory, and social aggression^9–13^. The oxytocin and vasopressin systems are key modulatory pathways involved in the control of lateral septum sociability^9,10,14–16^. Anatomically, the lateral septum is interconnected with a number of regions relevant for social behavior including the hippocampus, hypothalamus, periaqueductal gray, and medial amygdala^17^. Human imaging studies have indicated activation of the lateral septum with familial stimuli^18,19^. To address the role of the lateral septum in sibling-preference behavior, we first performed aspiration lesions of the lateral septum in young pups, which prefer siblings (<P13, Fig. 2a). Five pups were lesioned and tested for behavioral preferences a total of 53 times. All brains were histologically processed and assessed for lesion extent. Lesions avoided the medial septum (MS lesion area: 1.53 ± 0.56%) and on average lesioned 20.46 ± 8.36% of the lateral septum (Fig. 2b, c) in regions spanning the dorsal, intermediate, and ventral lateral septum (Supplementary Fig. 1a). Lesioned and non-lesioned pups were mixed and always tested in randomized order in the same testing session. Following aspirations of the lateral septum, the proportion of behavioral choices made by lesioned rats for siblings decreased and differed significantly from choices made by the non-lesioned <P13 population (<P13 data with no lesion, as in Fig. 1) when behavioral choices were pooled and when quantified on an individual pup performance basis (Fig. 2d, e). The behavioral performance of lesioned pups did not differ from 50/50 performance (<P13 LS lesion scores vs. 50/50 performance, Supplementary Table 1, *p* = 0.333, two-tailed Fisher’s exact test), however it should be noted that sibling-preference behavior reduced from 65 to 40% performance following lesions. Thus, it is possible that two independent mechanisms could be at play, one for kin preference and one a preference for novel rats. Further experiments such as testing preferences for cross-fostered siblings or unfamiliar genetic relatives could give further insight into this possibility.

### Lateral septum lesions disrupt non-sibling preference in old pups

**Fig. 3:**
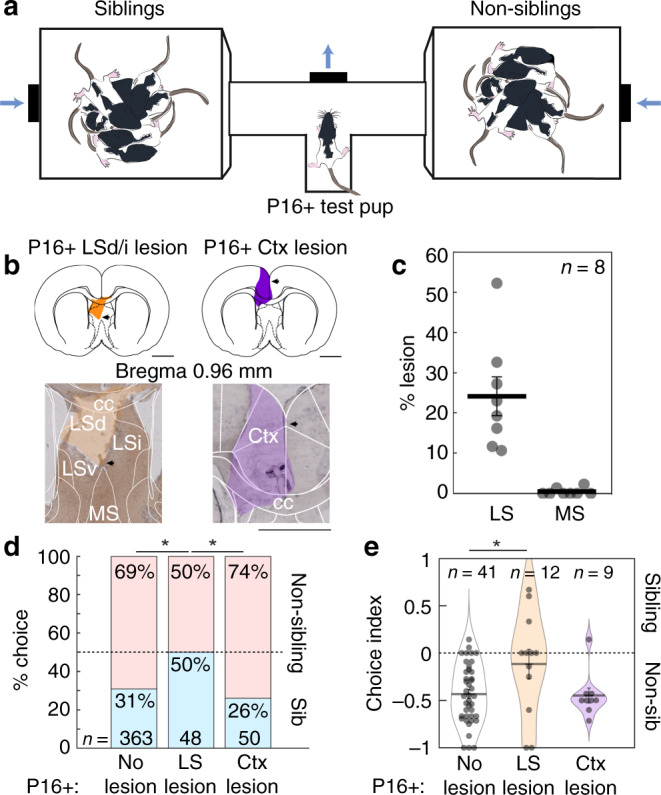
Non-sibling preference of old pups is disrupted by lesion of the lateral septum. **a** Apparatus for sibling-preference testing. Test pup was placed in the T-tube with two opposing chambers containing sibling and non-sibling rats. Air was blown toward the center of the T-choice tube. **b** Examples of two sections from two pups with lateral septal and cortical lesions (LS: lesion area in orange, Ctx: lesion area in purple). Scale bar applies to left and right micrographs. **c** Sections (100 µm thickness) of eight lesioned brains were analyzed for lesion extent. Four septal lesioned brains could not be measured due to damage during processing. Average percent lesion of lateral septum was estimated (LS: 24.09 ± 4.82%; MS: 0.46 ± 0.31%). Data represent the mean ± s.e.m. **d** Comparison of sibling preference in no lesion, lateral septum lesion and cortex lesion pups. Bars represent proportions of categorical choices pooled across animals (P16+ no lesion (as in Fig. 1d): 112 sibling choices, 251 non-sibling choices; P16+ lateral septal lesion: 24 sibling choices, 24 non-sibling choices; P16+ Ctx lesion: 13 sibling choices, 37 non-sibling choices; no lesion vs. LS lesion: *p* = 0.014; LS lesion vs. Ctx lesion: *p* = 0.021; no lesion vs. Ctx lesion: *p* = 0.517; Fisher’s exact test). **e** Sibling choice behavior by animals. Data represent the mean ± s.e.m. (No lesion vs. LS lesion: *p* = 0.012, *t*-test; no lesion vs. Ctx lesion: *p* = 0.893, *t*-test; LS lesion vs. Ctx lesion: *p* = 0.103, *t*-test; no lesion as in Fig. 1e). All tests are two-tailed. For detailed statistical information, see Supplementary Table 1. LSd dorsal lateral septum, LSi intermediate lateral septum, LSv ventral lateral septum, cc corpus callosum, MS medial septum, Ctx cortex. Scale bars: 2 mm. *p < 0.05.

We next addressed the role of the lateral septum in the sibling-avoiding age group (P16+). P16+ rats entered the center of the T-maze and turned towards their choice (sibling or non-sibling) direction (Fig. 3a). We performed lateral septum aspirations of 12 animals and control aspirations of the cortex overlying the lateral septal aspiration area of nine animals (Fig. 3b). Four lateral septum lesioned brains could not be measured due to damage during processing that could not be distinguished from lesion damage. The medial septum area was again avoided (MS lesion area: 0.46 ± 0.31%), while 24.09 ± 4.82% of the lateral septum was lesioned (Fig. 3b, c). The depth extent of the lateral septum lesioned brains spanned dorsal, intermediate, and ventral lateral septum regions and centered on the intermediate lateral septum area (Supplementary Fig. 1a). Following lateral septum lesions, sibling choices went to chance level (P16+ LS lesion scores vs. 50/50 performance, Supplementary Table 1, *p* = 1, two-tailed Fisher’s exact test) and significantly differed from choices of non-lesioned animals (Fig. 3d, e). In contrast, control aspirations of the overlying cortex did not impact the pooled sibling choice behavior or individual choice behavior of pups (Fig. 3d, e). Thus, our data support an essential role for the lateral septum in kin-preference and juvenile attachment behavior.

### Neuronal responses to kin stimuli in sibling-preferring young pups

**Fig. 4:**
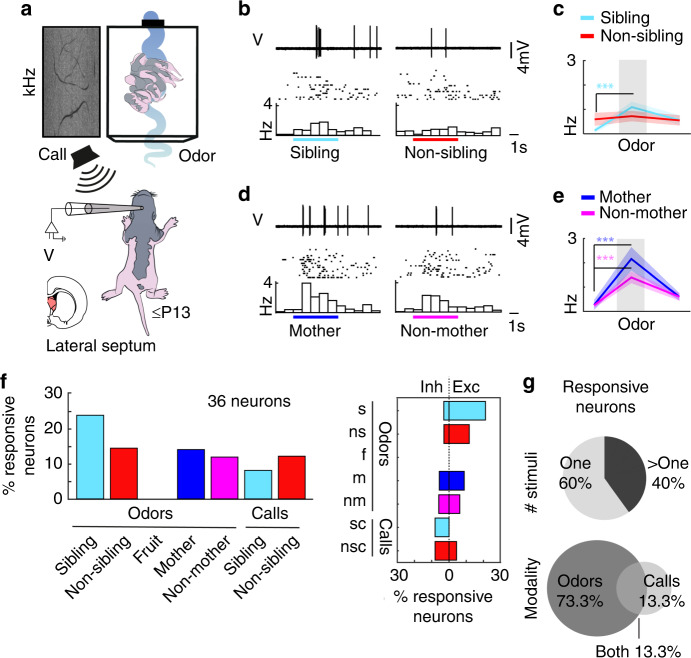
Responses of lateral septum neurons to kin stimuli in sibling-preferring young pups. **a** Juxta-cellular recordings of anaesthetized ≤P13 pups with odor and vocalization stimuli. **b** Responses to sibling and non-sibling odor (top), raster (middle), and peristimulus time histogram (bottom). Colored lines: timing of odors. **c** Firing rates for baseline (left), odor (middle), and offset (right). Data represent the mean (thick line) ± s.e.m. (shaded area). Sibling odor (*n* = 15 trials; mean ± s.e.m.; baseline = 0.13 ± 0.10 Hz, odor = 1.09 ± 0.22 Hz, difference = 0.96 ± 0.21; *p* = 4.0e−4, paired *t*-test) and non-sibling odor (*n* = 16 trials; mean ± s.e.m.; baseline = 0.59 ± 0.27 Hz; odor = 0.73 ± 0.21 Hz; difference = 0.13 ± 0.34; *p* = 0.705, paired *t*-test). **d** Conventions as in **b**. **e** Conventions as in **c**. Mother odor (*n* = 15 trials; baseline median = 0.00 Hz; odor median = 2.00 Hz; *p* = 3.7e−4, Wilcoxon signed rank test) and non-mother odor (*n* = 16 trials; mean ± s.e.m.; baseline = 0.25 ± 0.11 Hz; odor = 1.40 ± 0.24 Hz; difference = 1.15 ± 0.19; *p* = 2.2e−5, paired *t*-test). **f** Percent responsive neurons for each stimulus. Responsive neuron: significant change in firing rate within stimulus relative to baseline. Significance analyzed when trials ≥ 3. Left: Percent neurons responsive for each stimulus (*n* = responsive/total; sibling odor: *n* = 8/34, non-sibling odor: *n* = 5/35, fruit odor: *n* = 0/9, mother odor: *n* = 5/36, non-mother odor: *n* = 4/34, sibling call: *n* = 2/25, non-sibling call: *n* = 3/25). Right: Percent neurons inhibited or excited (*n* = inh:exc; sibling odor (s): *n* = 1:7; non-sibling odor (ns): *n* = 1:4; fruit (f): *n* = 0:0; mother odor (m): *n* = 2:3; non-mother odor (nm): *n* = 2:2; sibling call (sc): *n* = 2:0; non-sibling call (nsc): *n* = 2:1). **g** Of responsive neurons: 60.0% (9 neurons) responded to one stimulus and 40.0% (6 neurons) responded to multiple stimuli (top). 73.33% (11 neurons) responded to odors only, 13.33% (2 neurons) responded to only calls, and 13.33% (2 neurons) responded to calls and odors (bottom). All tests are two-tailed. For detailed statistical information, see Supplementary Table 1. ***p < 0.001.

To address the neural mechanisms underlying kinship behavior, we performed juxta-cellular and whole-cell patch-clamp recordings in the lateral septum of awake and anaesthetized rats in the sibling-preferring and sibling-avoiding age groups (≤P13 and P16+, respectively). Kin and non-kin odor stimuli consisted of siblings, non-siblings, the rat pup’s mother, and the mother from the non-kin litter. All stimulus animals were housed in a portable box with an electronically triggered fan that was activated for 5 s for each odor presentation. Prior to recordings, ultrasonic vocalizations of sibling pups and non-sibling pups were recorded and a 1 s segment was chosen when many rats emitted high-frequency vocalizations^20^. Once a recording was established, sibling, non-sibling, mother, and non-mother odors as well as sibling and non-sibling calls were presented repeatedly and the order of presentations was shuffled (Fig. 4a). In young pups (≤P13), we analyzed a total of 36 neurons. Neurons were distributed throughout the medial-lateral, anterior-posterior, and depth axis of the lateral septum (Supplementary Fig. 2a). In a subset of neurons where recordings could be maintained stably for repeated stimuli presentations, we observed consistent firing rate responses. Such responses, with preferential but not exclusive responses to subsets of social stimuli, predominated in the lateral septum. An example neuron, which responded significantly to sibling, mother, and non-mother odor presentations, is displayed (Fig. 4b–e). Across all neurons recorded where the number of stimulus presentations were three and greater, responsive neurons were observed for all social kin- and non-kin stimuli (Fig. 4f, left), with some neurons responding with inhibition and some with excitation of firing rates (Fig. 4f, right). Of the significantly responsive neuron population, 60% (nine neurons) responded to one stimulus and 40% (six neurons) responded to multiple stimuli (Fig. 4g, top), as seen in the example cell. In the young-pup age group, odor-responsive neurons predominated (73.3% odor responsive, 11 neurons) with 13.3% (2 neurons) responding significantly to calls and 13.3% (2 neurons) responding to both odors and calls (Fig. 4g, bottom).

### Neuronal responses to kinship stimuli in sibling-avoiding old pups

**Fig. 5:**
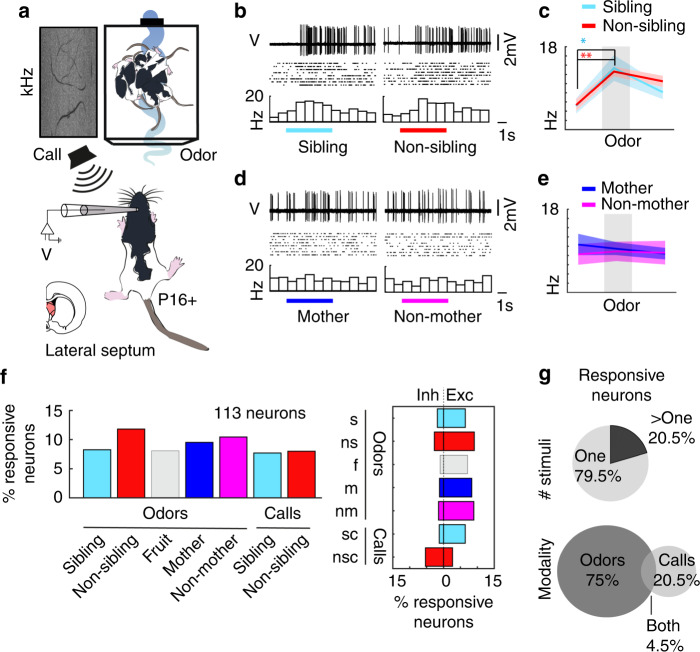
Responses of lateral septum neurons to kin stimuli in sibling-avoiding old pups. **a** Juxta-cellular recordings of awake and anaesthetized P16+ pups with odor and vocalization stimuli. **b** Responses to sibling and non-sibling odor (top), raster (middle), and peristimulus time histogram (bottom). Colored lines: timing of odors. **c** Firing rates for baseline (left), odor (middle), and offset (right). Data represent the mean (thick line) ± s.e.m. (shaded area). Sibling odor (*n* = 8 trials; mean ± s.e.m.; baseline = 5.13 ± 1.64 Hz; odor = 13.38 ± 2.80 Hz; difference = 8.25 ± 3.22; *p* = 0.038, paired *t*-test). Non-sibling odor (*n* = 8 trials; mean ± s.e.m.; baseline = 5.31 ± 1.29 Hz; odor = 12.63 ± 1.04 Hz; difference = 7.31 ± 1.55; *p* = 0.002, paired *t*-test). **d** Conventions as in **b**. **e** Conventions as in **c**. Mother odor (*n* = 8 trials; mean ± s.e.m.; baseline = 10.38 ± 2.29 Hz; odor = 9.48 ± 1.38 Hz, difference = 0.90 ± 1.43; *p* = 0.55, paired *t*-test). Non-mother odor (*n* = 8 trials; mean ± s.e.m.; baseline = 8.25 ± 2.25 Hz; odor = 8.88 ± 2.07 Hz; difference = 0.63 ± 1.76; *p* = 0.733, paired *t*-test). **f** Percent responsive neurons for each stimulus. Responsive neurons: significant change in firing rate within stimulus relative to baseline. Significance analyzed when trials ≥ 3. Left: Percent neurons responsive for each stimulus (*n* = responsive:total neurons; sibling odor = 9:109, non-sibling odor = 13:110, fruit odor = 8:99, mother odor = 8:84, non-mother odor = 7:67, sibling call = 6:78, non-sibling call: 6:75). Right: Percent neurons inhibited or excited (*n* = inh:exc; sibling odor (s) = 2:7; non-sibling odor (ns) = 3:10; fruit (f) = 1:7; mother odor (m) = 1:7; non-mother odor (nm) = 1:6; sibling call (sc) =  1:5; non-sibling call (nsc) = 4:2). **g** Of responsive neurons, 79.5% (35 neurons) responded to one stimulus, and 20.5% (9 neurons) responded to multiple stimuli (top). 75.0% (33 neurons) responded to odors only, 20.5% (9 neurons) responded to calls only, and 4.5% (2 neurons) responded to calls and odors. All tests are two-tailed. For detailed statistical information, see Supplementary Table 1. *p < 0.05, **p < 0.01.

We previously showed that kinship behavior changes drastically in juvenile pups, with a switch occurring around 15 postnatal days of age, thus we next made recordings from pups of the sibling-avoiding age group (P16+) with kin- and non-kin stimuli (Fig. 5a). The analysis of firing rate responses consisted of 113 neurons (28 awake recordings, 85 recordings under anesthesia), which were similarly spread across all axes of the lateral septal area (Supplementary Fig. 2b). Neurons recorded from pups in the P16+ age group responded to social kin-, non-kin, and non-social fruit stimuli. An example cell responded to sibling odor and non-sibling odor and not to mother and non-mother odors (Fig. 5b–e), thus, this cell did not discern kinship (kin vs. non-kin stimuli) based on firing rate changes. The total proportion of lateral septal neurons showing significant suprathreshold odor and vocalization responses was in the range of 10% (Fig. 5f, left). Neurons responded with inhibition and excitation of firing rates to all stimuli (Fig. 5f, right). Responses to ultrasonic vocalizations often resulted in a reduction of suprathreshold firing (Fig. 5f, Supplementary Fig. 3a). The majority of neurons responded significantly to a single stimulus (single stimulus: 79.5%, 35 neurons; multiple stimulus: 20.5%, 9 neurons; Fig. 5g, top). A larger proportion of neurons responded to odors (75%, 33 neurons) compared with calls (20.5%, 9 neurons) and few neurons (4.5%, 2 neurons) responded to both odors and calls (Fig. 5g, bottom). Thus, neurons of the lateral septum respond broadly to kin- and non-kin multisensory stimuli and neurons responsive to kin odors and kin vocalizations appear to be largely non-overlapping, indicating that auditory and odor information may be routed to the lateral septum in distinct pathways.

### Subthreshold responses of lateral septum neurons to kin stimuli

**Fig. 6:**
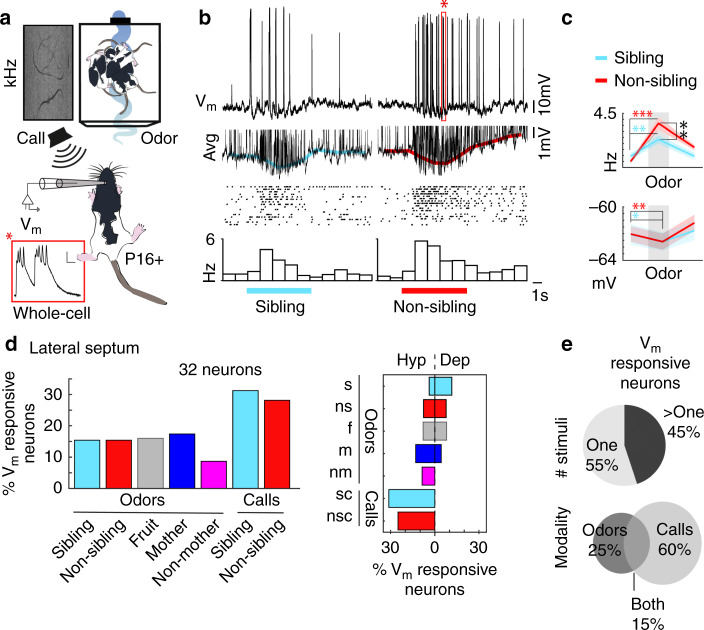
Subthreshold responses of lateral septum neurons to kin stimuli. **a** Patch-clamp recordings of anaesthetized P16+ pups with odor and vocalization stimuli. Inset: plateau potential, scale bar: 10 mV/10 ms, from example in **b** (star). **b** Top: Membrane potential (V_m_) example; below: average V_m_ across trials, colored lines: average V_m_ after spike removal; middle: raster; bottom: peristimulus time histogram. Colored lines: timing of odor. **c** Top: Spike rate, sibling odor (*n* = 16 trials; mean ± s.e.m.; baseline = 0.94 ± 0.25 Hz; odor = 2.34 ± 0.35 Hz; difference = 1.40 ± 0.44; *p* = 0.006, paired *t*-test), and non-sibling odor (*n* = 16 trials; mean ± s.e.m.; baseline = 0.50 ± 0.13 Hz; odor = 3.66 ± 0.51 Hz; difference = 3.16 ± 0.47; *p* = 6.7e−6, paired *t*-test). Rate difference: sibling vs. non-sibling (*n* = 16 trials; difference mean ± s.e.m. = 1.33 ± 0.43; *p* = 0.007, paired *t*-test). Bottom: V_m_ response sibling odor (*n* = 16 trials; mean ± s.e.m.; baseline = −62.06 ± 0.79 mV; odor = −62.59 ± 0.66 mV; difference = 0.53 ± 0.21; *p* = 0.021, paired *t*-test), and non-sibling odor (*n* = 16 trials; mean ± s.e.m.; baseline = −62.03 ± 0.58 mV, odor = −62.60 ± 0.60 mV, difference = 0.57 ± 0.18; *p* = 0.007, paired *t*-test). **d** Left: Percent V_m_ responsive neurons. Responsive neurons: Significant change in V_m_ with stimulus relative to baseline. Analyzed with trials ≥ 3. Percent V_m_ responsive neurons for each stimulus (*n* = responsive:total neurons; sibling odor = 4:26, non-sibling odor: 4:26, fruit odor: 4:25, mother odor: 4:23, non-mother odor: 2:23, sibling call: 2:32, non-sibling call: 9:32). Right: Percent neurons with hyperpolarization or depolarization (*n* = hyp:dep; sibling odor (s) = 1:3; non-sibling odor (ns) = 2:2; fruit (f) = 2:2; mother odor (m) = 3:1; non-mother odor (nm) = 2:0; sibling call (sc) = 10:0; non-sibling call (nsc) = 8:0). **e** Of V_m_ responsive neurons, 55.0% (11 neurons) responded to one, and 45% (9 neurons) to multiple stimuli (top). 25.0% (5 neurons) responded to odors only, 60.0% (12 neurons) to calls only, and 15% (3 neurons) to calls and odors (bottom). All tests are two-tailed. For detailed statistical information, see Supplementary Table 1. *p < 0.05, **p < 0.01, ***p < 0.001.

We next asked how kin stimuli are integrated in the subthreshold activity of lateral septal neurons. We analyzed subthreshold responses of 32 neurons from sibling-avoiding P16+ pups. Whole-cell patch-clamp recordings with kin and non-kin stimuli revealed a complex array of subthreshold events consisting of highly varied postsynaptic potential amplitudes, spikelets, and plateau potentials (Fig. 6a, b, inset). In the example neuron, these diverse response components interacted in a complex way, leading to an excitatory suprathreshold response riding on an overall membrane potential hyperpolarization. In this particular cell, we observed a statistically significant difference between the responses to kin- and non-kin stimuli (Fig. 6b, c). Across the population of neurons recorded, subthreshold responses to kin-odor and vocalization stimuli were observed in subthreshold activity. The response selectivity of individual neurons was again heterogeneous, where certain cells showed responses to calls and others to odors. A larger proportion of neurons responded with subthreshold changes in the membrane potential than with firing rate analysis (in the range of 15–30% for each of the stimuli, Fig. 6d, left), subthreshold responses consisted of both hyperpolarization and depolarization of the membrane potential (Fig. 6d, right), and neurons responded to single and multiple stimuli (55% one stimuli subthreshold, 45% more than one stimuli subthreshold, Fig. 6e, top). The proportion of neurons responding to sensory modalities was distinct compared with what was observed in suprathreshold activity, where we found more subthreshold call-responsive neurons (60%, 12 neurons) compared with odors (25%, 5 neurons) and 3 neurons responded to both (15%, Fig. 6e, bottom). Subthreshold call-responsive cells were predominantly hyperpolarized in response to ultrasonic vocalization call playback (Supplementary Fig. 3b), which is likely a mechanism for suprathreshold inhibition of firing rate responses to calls (Fig. 5, Supplementary Fig. 3a). Across all age groups and recording configurations, relatively few of the significantly responding neurons responded to both odor and call stimuli (13.3% P0–13, 4.5% P16+ suprathreshold, 15% subthreshold), thus we plotted estimated locations of call and odor-responsive cells across the axes of the lateral septum for the P16+ firing rate dataset. Although the population of odor and call-responsive neurons were distinct, we did not observe a clear anatomical organization (dorso-ventral, lateral-medial, or anterior-posterior) according to sensory modality (Supplementary Fig. 4a, b). Overall, these physiological observations show: that responses to multimodal social stimuli are common in the lateral septum throughout development; that narrowly tuned suprathreshold AP responses (Figs. 4, 5) are generated from broadly tuned synaptic inputs evident in the membrane potential responses (Fig. 6); and that integration of social sensory modalities occurs in distinct (odor and vocalization responsive) subsets of neurons that are intermingled anatomically across the axes of the lateral septum.

### Development of ongoing activity and proportion of kin responsive neurons

**Fig. 7:**
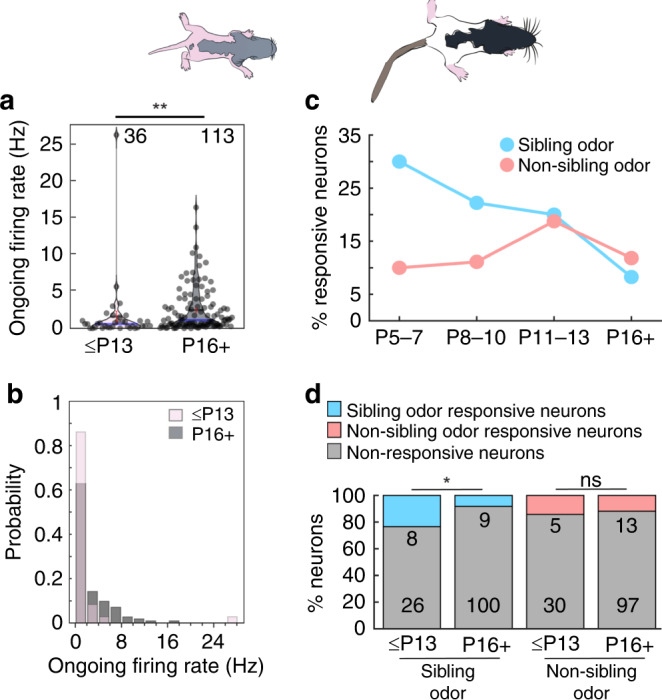
Development of ongoing firing and kin-odor-responsive neurons in the lateral septum. **a** Ongoing firing rate was lower in young vs. old pups (≤P13: *n* = 36; median = 0.59 Hz; mean ± s.e.m. = 1.59 ± 0.73 Hz; P16+: *n* = 113; median = 1.12; mean ± s.e.m. = 2.46 ± 0.29 Hz; difference of medians = 0.53 Hz; *p* = 0.004, Mann-Whitney rank sum test). Horizontal blue line is the median, horizontal black line is the mean. Error bars are s.e.m. **b** Probability histogram of ongoing firing rates (bins range from 0 to 30 in 2 Hz increments). **c** Percentage of significantly suprathreshold responsive neurons with developmental age. As previously described, significance for stimuli was analyzed when number of trials was ≥3. Sibling odor significant neurons (*n* = responsive:total neurons; P5–7 = 3:10 neurons, P8–10 = 2:9 neurons, P11–13 = 3:15 neurons, P16+ = 9:109 neurons). Non-sibling odor significant neurons (*n* = responsive:total neurons; P5–7 = 1:10 neurons, P8–10 = 1:9 neurons, P11–13 = 3:16 neurons, P16+ = 13:110 neurons). **d** Proportion of suprathreshold odor significant and odor non-significant neurons for sibling odors and non-sibling odors in sibling-preferring and sibling-avoiding age groups. ≤P13 (sibling odor: 8 significant, 26 non-significant; non-sibling odor: 5 significant, 30 non-significant), P16+ (sibling odor: 9 significant, 100 non-significant; non-sibling odor: 13 significant, 97 non-significant). Proportion of sibling odor-responsive neurons was different between age groups (≤P13 vs. P16+ sibling odor responsive; *p* = 0.029, Fisher’s exact test) and not different for proportion of non-sibling-responsive neurons (≤P13 vs. P16+ non-sibling odor responsive; *p* = 0.77, Fisher’s exact test). Colored bars represent proportion of significantly responsive sibling or non-sibling neurons. Gray areas indicate proportion of neurons which were not significantly responsive. All tests are two-tailed. For detailed statistical information, see Supplementary Table 1. *p < 0.05, **p < 0.01, ns not significant.

Although the tuning of lateral septal cells in young sibling-approaching and older sibling-avoiding pups appears to be broad in both cases, some differences exist. We noted that ongoing firing rates of all recorded cells undergo an age-dependent increase (correlation coefficient with age = 0.316; *p* = 8.6e−5, Spearman rank order correlation) where lateral septal neurons in young ages have lower ongoing firing (Fig. 7a, b). We additionally found that the proportion of suprathreshold sibling odor-responsive cells in the young pup age group was larger. We binned the proportion of responsive cells with developmental age and saw the largest proportion of sibling-responsive cells in the youngest (P5–7) age group, which declined with age (Fig. 7c). When we grouped the proportion of sibling odor and non-sibling odor-responsive cells across the two age groups (≤P13 and P16+), a significant difference was observed in the proportion of sibling odor-responsive cells, but not in the non-sibling odor-responsive cell proportions (Fig. 7d). Thus, we find that resting excitability, indicated by ongoing firing rate, as well as social response selectivity change with age. The observed developmental changes in kinship behavior may be supported by age-dependent changes in cellular response-tuning for kin-odor stimuli.

### Nepotopy of kin responsive neurons in the lateral septum

**Fig. 8:**
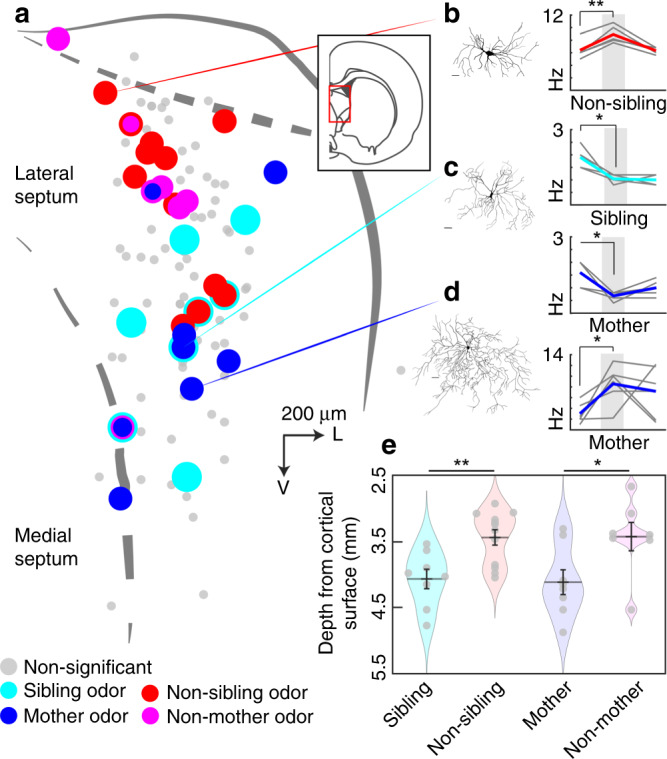
The lateral septum shows nepotopy—a representation of response preferences ordered according to kinship. **a** Locations of recorded neurons from P16+ age group with significant responses to kin- and non-kin stimuli color coded. See “Methods” for details on how this composite map of cells was plotted. V ventral, L lateral, scale bar = 200 µm. **b** Left, morphological reconstruction of dorsal lateral septal neuron responding to non-sibling odors. Right, firing rates in baseline (left), during odor presentation (middle) and the offset period (right) for non-sibling odor (*n* = 5 trials; baseline mean ± s.e.m. = 6.40 ± 0.68 Hz; odor mean 8.92 ± 0.63 Hz; difference = 2.52 ± 0.39 Hz; *p* = 0.003, paired *t*-test). **c** Left, morphological reconstruction of lateral septum neuron significantly inhibited by mother and sibling odors. Sibling odor (*n* = 5 trials; baseline mean ± s.e.m. = 1.90 ± 0.19 Hz; odor mean 1.04 ± 0.08 Hz, difference = 0.86 Hz; p = 0.024, paired *t*-test). Mother odor (n=5 trials; baseline mean 1.60 ± 0.25 Hz; odor mean 0.68 ± 0.08 Hz; difference = 0.92 ± 0.27 Hz; *p* = 0.026, paired *t*-test). **d** Left, morphological reconstruction of a lateral septum neuron responding significantly to mother odor (*n* = 6 trials; baseline mean 3.00 ± 0.76 Hz; odor mean 8.57 ± 1.48 Hz; difference = 5.57 ± 1.62 Hz; *p* = 0.019, paired *t*-test). **e** Depths of significantly responsive cells. Sibling odor responsive cells (*n* = 8; mean depth ± s.e.m. = 4068 ± 148 µm). Non-sibling odor responsive (*n* = 12; mean depth ± s.e.m. = 3441 µm ± 115 µm). Sibling vs. non-sibling depth comparison (difference = 627.125 µm; *p* = 0.003, *t*-test). Mother odor responsive neurons (*n* = 8; mean depth ± s.e.m. = 4117 ± 532 µm). Non-mother odor responsive cells (*n* = 7; mean depth ± s.e.m. = 3429 ± 567 µm). Mother vs. non-mother depth comparison (difference = 688.036 µm; *p* = 0.031, *t*-test). Thick black horizontal lines represent means, error bars are s.e.m. Thick colored lines represent the mean of firing rates and gray lines in the background represent individual trials. All tests are two-tailed. Scale bars for reconstructed cells: 25 µm. For detailed statistical information, see Supplementary Table 1. *p < 0.05, **p < 0.01.

Lateral septum neurons respond preferentially, but not exclusively, to social stimuli and few neurons appear to discriminate between kin- and non-kin stimuli in their firing rate and membrane potential responses. This poses the question: how is the neural code for kinship organized from such non-specific responses? We turned to the anatomical locations of cells we recorded with post-recording histology for confirmation of cellular locations. We plotted the coordinates of all neurons for which we assessed response preferences and recovered coordinate information from our most substantial dataset (P16+ suprathreshold, 109 neurons, 4 neurons without coordinate information). Neurons, which responded significantly to non-sibling odor stimuli or non-mother stimuli, were located more superficially in the lateral septum (Fig. 8a, e). One such superficially located neuron that responded significantly to non-sibling odors is shown in Fig. 8b. Sibling and mother-responsive neurons were located more ventral in the lateral septum (Fig. 8a, e). One such more ventral neuron (Fig. 8c, left) showed significant inhibition for both mother odor stimuli (Fig. 8c, bottom right) and sibling odor stimuli (Fig. 8c, top right). Another more ventral neuron was significantly activated for odor stimuli from the pup’s mother (Fig. 8d). Despite clear morphological differences in the reconstructed neurons, the number of reconstructed cells, at this point, is not enough to provide a quantitative comparison. We found that sibling-responsive neurons were located significantly more ventral than non-sibling-responsive neurons and found that mother-responsive neurons were located significantly more ventral than non-mother-responsive neurons (Fig. 8e). We conclude that response preferences in the lateral septum are ordered according to kinship and such spatial order could establish a functional topographic code for kinship by allowing the selective routing of kin- and non-kin-preferring responses. We refer to this organization as nepotopy (Latin: *nepos*, nephew; Greek: *topos*, place).

## Discussion

We confirm the existence of elaborate and age-dependent kin preferences in rats and have shown that lateral septum lesions abolish such preferences. Neuronal responses in rat lateral septum show complex and multisensory responses to social stimuli, age-dependence of ongoing activity, and the proportion of kin responsive cells as well as marked dorso-ventral differences according to kinship, an organization we refer to as nepotopy. Our data identify the lateral septum as a neural substrate of kinship behavior. The nepotopy of the lateral septum could provide a mechanistic framework for how the brain computes social identity and generates behavior based on kin preferences.

This work provides the first evidence of a neural correlate for kinship behavior. Social attachment to genetic relatives is widespread and prevalent and mechanisms of kin recognition are widely debated in the fields of behavioral science and evolutionary theory, however fundamental questions about how these bonds are formed and change over time have so far remained unanswered. Is kinship and attachment to genetic relatives innate or is it a mechanism that is learned from sensory experience? Work by Hepper, demonstrating a preference of newborn pups for their mother’s amniotic fluid, suggests that kinship preference is present at birth^8^, however this does not exclude the possibility that kin memory is subject to fetal learning mechanisms. In support of this are findings that multisensory associations are formed readily in the fetus^21^. Rat pups can learn olfactory associations in utero, for example, as demonstrated by behavioral analysis following injection of mint solution in the amniotic fluid^22^. Further evidence supports the possibility that kinship behavior may undergo experience dependent changes. Experiments with cross-fostering of rat pups from birth demonstrate the development of preference for cross-fostered siblings over stranger pups^7^. Can nepotopy, for example, be established in the lateral septum from learned familiarity or is it a representation that pre-dates such learning processes? We have demonstrated that an intact lateral septum is necessary for sibling-preference behavior and that the neurons of the lateral septum are responsive to multisensory kinship stimuli, thus providing a strong framework for further exploration of how the brain may learn and represent kinship.

The lateral septum receives input from the hippocampus in a dorso-ventral organization^17^, which may underlie the selective responsiveness to odor stimuli to kin in the intermediate region. The CA2 region of the dorsal portion of the hippocampus and the ventral hippocampus in general have been recognized for their role in social memory^23–25^. Hippocampal inputs to the lateral septum, thus, may activate neurons in the intermediate lateral septum following social stimuli. A circuit between the hippocampus, lateral septum, and hypothalamus has been shown to modulate social aggression in adult rodents^26,27^, lateral septal vasopressin and oxytocin systems modulate social memory in males^14^ and social fear in lactating females^9^, and lateral septal vasopressin antagonists modulate juvenile play in a sexually dimorphic manner^28^. Our study provides an ethologically relevant interface for these and other studies that dissect the role of the lateral septum in specific aspects of social behavior. Kinship behavior requires numerous stages of processing starting from memory recall and ending with selection of the appropriate action such as aggression, mating, and/or attachments. Simultaneous recordings in the hippocampus and lateral septum from initial social approach to action selection will be informative in elucidating the order and nature of social information flow which supports a broader circuitry of kinship behavior.

Deficits in social communication and disturbances in natural juvenile social behaviors such as play are present in autism spectrum disorders (ASD)^29^. Study of the fundamental neural mechanisms of the earliest and strongest social relationship, those with kin, will provide insights into how the brain supports the development of social communication and attachments and how they are disrupted in ASD. A unique set of hypotheses emerge from the present study, including whether ordered topographies related to kinship and familiarity are established and transformed in a disparate manner in diseased brains. How does the ASD brain respond to multisensory social stimuli? How kin-responsive lateral septum neurons are modulated with age is another important question. The mechanism for the observed developmental changes might be mediated by changes in oxytocin and vasopressin receptor expression^26,27^, which could support changes in neuronal excitability and/or modulation excitation/inhibition balance of sensory processing^30–32^. Excitation/inhibition imbalance is documented in animal models of ASD^33^. The observed developmental change in lateral septum ongoing activity and proportions of kin-responsive neurons could provide insights into how these developmental trajectories might differ in models of ASD. One interesting and related hypothesis is a role for endogenous opioids in social attachment behavior^34,35^. The intermediate lateral septum receives input from the leu-enkephalin fibers of the hypothalamus and the medial amygdala^17^ and forms output to areas relevant for social and sexual behaviors such as the lordosis reflex in the periaqueductal gray^36^. We suggest that the lateral septum is not only a substrate for pair bonding, as previously shown^37^, but that it also plays a key role in kin-affiliation as indicated by the present study and human imaging studies^18,19^. Our work provides a neural substrate of kinship behavior and has implications for understanding how mate selection, altruistic, and attachment behaviors are computed at the circuit and synaptic level.

## Methods

### Animals and behavioral testing

Male and female Long-Evans rats age P2–30 were used in this study. A total of 77 rats were used for lesion, non-invasive behavior and electrophysiological experiments. In some cases, rats used for lesion or electrophysiology experiments were first tested for non-lesion behavior. Experimental procedures were performed according to the guidelines of the local ethics committees: German guidelines on animal welfare under the supervision of the local ethics committees (Landesamt für Gesundheit und Soziales: G0279/18) and the Animal Care and Use Committees at the Shenzhen Institute of Advanced Technology (SIAT), Chinese Academy of Sciences (CAS), China (SIAT-IRB-171016-NS-WH-A0384).

Kin-preference testing apparatus were constructed based on the guidelines published by Hepper^7^. As diagrammed (Figs. 1a, 2a, and 3a), a T-tube made of plastic was connected to two chambers containing either the sibling litter or the non-sibling litter of the tested rat. To control for the possibility that rats may have learned the spatial location or used cues from the room to orient themselves, the location of the test boxes was alternated between tests and the apparatus was rotated regularly. In between each rat tested, the T-arms were cleaned with dilute 70% ethanol and dilute bleach. At young ages when the rat’s crawling ability was impaired, choices were scored based on the direction that the rat turned into the T-arm, either indicated by a clear head and/or body turn. As rats matured, the size of the T-arms were enlarged, also in line with the methods of Hepper^7^. When the rat’s crawling ability was fully matured, choice behavior was scored by the side of the T-arm where the rat first fully crawled to the wire mesh separating the T-arm and the litter-containing box, thus displaying interest in social interaction with the respective litter. Tests were performed one to two times per day with a separation of morning and afternoon between testing periods. Lesioned pups were given a minimum of 4 h to recover before testing. In an additional series of experiments, choice behavior was tested following isoflurane anesthesia and we confirmed that the anesthesia did not affect choice performance when rats were tested 3–4 h after being anaesthetized. The choice index for each pup was computed

1 $${\mathrm{choice}}\,{\mathrm{index}} = \frac{{\left( {{\sum} {{\mathrm{sibling}}\,{\mathrm{choices}} - {\sum} {{\mathrm{nonsibling}}\,{\mathrm{choices}}} } } \right)}}{{\left( {{\sum} {{\mathrm{sibling}}\,{\mathrm{choices}} + {\sum} {{\mathrm{nonsibling}}\,{\mathrm{choices}}} } } \right)}}.$$

### Lesions

Aspiration lesions were performed under hypothermia (up to age P5) or isoflurane anesthesia. Following anesthesia of the animal, lidocaine (2%) was injected locally and skin was incised and then a craniotomy was drilled in the skull 1–2 mm in diameter centered around +0.5 mm anterior and 1 mm lateral of bregma. The dura was then opened in a 1–1.5 mm area and a glass pipette with an opening of ~200–400 μm diameter connected to an electric vacuum pump was used to aspirate either the control cortical or cortical and lateral septum area. The aspiration area was visually guided using a stereomicroscope. Following completion of the aspiration, the incised skin area was closed with dissolvable sutures and/or Vetbond tissue adhesive. Lesioned animals were checked for health status multiple times daily. After completion of behavioral testing, lesioned animals were perfused with 4% paraformaldehyde (PFA) and brains were stored overnight. Brains were then sectioned at 100 μm thickness, processed with cytochrome oxidase staining, and then mounted on microscope slides.

Using an MBF CX9000 (Optronics, Goleta, CA) camera with Neurolucida software (MBF Bioscience, Williston, VT, USA) and ImageJ software, lesioned areas were traced and quantified. Area per section was multiplied by section thickness (100 μm) to estimate volume. Total volume per animal was summed across sections. Percent of lateral septum and medial septum areas were estimated by tracing the entire lateral and medial septal area across sections and the lesioned volume was divided by the estimated intact region volume. Relative lesion depths were estimated by measuring the upper and lower boundary of the lateral septum for each section, then measuring the lower (most ventral) extent of each lesion area. The lower lesion extent was then divided by the height of the lateral septum for that section. Each section then had a value for relative lesion depth, 0 corresponding to the most superficial part, and 1 corresponding to the deepest. This was averaged across all sections to estimate the relative lesion depth per animal (Supplementary Fig. 1). Four of the twelve P16+ pup lesioned brains were not analyzed for lesion extent due to damage in histological processing that could not be distinguished from lesion damage.

### Electrophysiological recordings

Methods for in vivo juxta-cellular and whole-cell patch-clamp recordings were similar to those previously published^38^. Juxta-cellular and whole-cell patch recordings were amplified (Dagan BVC-700A, Dagan, Minneapolis, MN), low-pass filtered at 10 kHz, and sampled at 50 kHz by a data-acquisition interface (Power 1401, CED, Cambridge, England) controlled by Spike2 software (CED, Cambridge, England). Pipettes were filled with an internal solution containing the following (in mM): K-gluconate 135, HEPES 10, phosphocreatine-Na 10, KCl 4, ATP-Mg 4, GTP 0.3, and pH 7.2. The craniotomy for all recording experiments was made by drilling either a 2 mm (medial-lateral) by 3 mm (anterior-posterior) bilateral square, which was centered on 0.5 mm anterior and 0 mm lateral to bregma, or a unilateral square extending from a perimeter just adjacent to the midline to 1.5 mm lateral and an anterior-posterior perimeter extending from 1.5 mm anterior to 1 mm posterior.

Prior to electrophysiological recordings, ultrasonic vocalizations of sibling and non-sibling rats were recorded using Avisoft UltraSoundGate 416H system with ultrasonic microphones and Avisoft Recorder USGH software. During recording periods, only the litter of interest was in the room. Vocalizations were recorded for time periods of 20–60 min and a 1-s period of vocalizations were chosen to use as stimuli. The vocalization stimuli were chosen when multiple rats from the litter were vocalizing, which was apparent by multiple overlapping vocalizations and when vocalizations were in the high-frequency vocalization range, suggesting positive affect of the litter^20^. Odor stimuli for electrophysiological recordings consisted of the sibling litter, non-sibling litter, mother, non-mother animals, and fruit odor stimuli (fresh lemon, apple, and orange), which were housed in a plastic enclosed box with an opening covered by wire mesh at the front of the box (7.5 cm in diameter). On the opposing side of the opening, another opening with wire mesh was made in the box (10.2 cm^2^) with a TTL-triggered computer fan attached (Pure Wings 2, 120 mm). All boxes were constructed identically and were ensured to emit equal amounts of air-flow upon triggering.

Acute juxta-cellular recordings were performed under urethane anesthesia (1.5–2 g/kg). Chronic awake juxta-cellular recordings proceeded as follows: juvenile animals (male and female) were habituated by handling prior to surgical procedures for 2–3 days. Anesthesia consisted of an initial injection of 100 mg/kg ketamine and 7.5 mg/kg xylazine or isoflurane (0–4% in O_2_) anesthesia. Reflexes (respiration, blink, and pinch response) were monitored throughout the procedure and an extra dose of ketamine alone (25% of initial dose) was given as needed. Temperature was maintained with a heating pad (Stoelting) set to 34–36 °C and monitored using a rectal probe. Lidocaine was locally injected under the scalp prior to removal. In the first surgery, the skull was thoroughly cleaned and a metal bolt was implanted using UV hardening glue (Kerr) and dental cement (Heraeus). After successful habituation of the implanted subject rat to head fixation, the craniotomy was made (details above). Dura was left intact for awake juxta-cellular recordings and the preparation was protected by implantation of a threaded plastic cylinder with removable lid. The preparation was covered using silicone in between recording sessions (Kwik-Cast, World Precision Instruments).

The search procedure for juxta-cellular recordings proceeded with lowering of the patch pipette into the brain (Grass Technologies AM10) while steps were made in 4 µm increments with a micromanipulator (Luigs & Neumann SM-5). Spike activity was monitored using an audio monitor (Grass Technologies AM10). When activity was detected, electrophysiological recording and video recordings of social presentations began. As long as the preparation permitted further recordings, sessions were performed daily.

In vivo patch-clamp recordings^39^ were performed in juvenile rats age P20–30 under urethane anesthesia (1.5–2 g/kg). Positive pressure was applied (200–300 bars) and pipettes were lowered into the brain. When the pipette reached ~2000 µm below the surface, pressure was reduced to 20–30 bars for cell searching. Resistance of patch pipettes ranged from 4 to 6 MΩ. Pipette resistance was monitored online with stepping procedures. When the pipette resistance increased, pressure was released and suction, if necessary, was applied to establish a gigaohm seal. Break-in was performed with brief mouth-applied suction to establish whole-cell configuration. Upon establishment of a whole-cell recording, several seconds of spontaneous activity were recorded. Pre-recorded sibling or non-sibling vocalizations, sibling, non-sibling, mother, and non-mother odors were then presented.

In the final day of chronic and in acute in vivo experiments neurobiotin (0.01–1%, Vector) was added to the pipette solution for post-hoc analysis of the recorded cells. In juxta-cellular recording configuration, the Pinault technique^40^ was used to label recorded neurons. After the in vivo recording session, animals were anaesthetized using urethane anesthesia (1.5–2 g/kg) and perfused with phosphate buffer followed by a 4% PFA solution. Brains were stored overnight in 4% PFA before performing 100 µm coronal sectioning. Neurobiotin-filled neurons were visualized using fluorescent streptdavidin (1:1000, Invitrogen streptavidin, Alexa 488). Sections were visualized using a Leica epifluorescence microscope.

### Mapping of neuron coordinates

Coordinates for all recordings were recorded by zeroing the pipette location at the center of bregma and at the depth of the top of the brain before entering using a micromanipulator (Luigs & Neumann SM-5). Once a juxta-cellular or whole-cell recording was completed, the anterior-posterior, medial-lateral, and depth of the cell was recorded. At the end of data collection and after histological processing of brains, mounted sections were imaged with fluorescence microscopy to locate the labeled cells. Cells were assigned based on the pre-recorded coordinates and location relative to other cells in the recorded brain. We recovered, assigned, and measured the coordinates of 34 labeled cells. Accuracy of the recorded micromanipulator coordinates was assessed by comparing anatomically identified coordinates with those recorded from the micromanipulator. We determined the median difference of coordinates recorded with the micromanipulator vs. those measured anatomically (anterior coordinates difference, median = 200 µm; lateral coordinates difference, median = 23 µm; depth coordinates difference, median = 125 µm) and adjusted coordinates of anatomically un-assigned cells accordingly. Assignment of cells was performed blind to the results of the experiment.

To plot a composite map of cells and to perform depth comparisons between sibling/non-sibling and mother/non-mother responses, we superimposed all cells in one coordinate frame using the coordinate alignment procedures described above. We then plotted and color coded the position of all significant sibling/non-sibling and mother/non-mother responses. In cases where a cell responded significantly to both sibling and non-sibling or mother and non-mother stimuli, we performed a statistical comparison between these responses. If there was a statistically significant difference between sibling/non-sibling or mother/non-mother within-odor responses, we plotted the color of the preferred response in Fig. 8a and included only the preferred response in Fig. 8e. The outline of the lateral septum in the composite map shown in Fig. 8a was chosen by aligning drawings of five sections with histologically recovered lateral septum neurons to the top medial point of the lateral septum. We superimposed the composite map on the lateral septum outline that ran in the middle of these five outlines. Example cells were reconstructed using a MBF CX9000 (Optronics, Goleta, CA) camera using Neurolucida software (MBF Bioscience, Williston, VT, USA). Multiple z-stack images were first collected using a Leica Epifluorescence microscope and z-stacks were aligned in the Neurolucida software.

### Electrophysiological analysis and statistics

Electrophysiological data were analyzed using Spike2 (CED, Cambridge, England) and Matlab 2019b (MathWorks, Natick, MA) software. Firing rate and subthreshold activity was acquired continuously for each respective cell. Spike times were detected by DC filtering the recording and setting a threshold just above the noise. In patch-clamp and juxta-cellular recordings from lateral septal neurons, we observed more heterogeneous spike amplitudes and spikelet events compared with what was previously observed in cortical recordings. When such graded amplitude spikes and spikelets were present, these events were included in the analysis (such as in the Fig. 6 example). Parameters for spike detection were set once and applied across the entire continuous recording period and all stimulus presentations for that neuron. All neurons analyzed for suprathreshold properties exhibited spontaneous spikes in the search period and/or initial recording period prior to the presentation of stimuli. Thus, in whole-cell recordings where spontaneous firing was not present in the search period were not analyzed for suprathreshold properties. Initiation times of vocalization play and the start of the fan for each respective odor were TTL triggered in the recording and the trigger times were aligned with spike times and membrane potential in Matlab. The baseline period for vocalization and odor stimuli was a 2 s period prior to the stimulus trigger. Within stimulus periods were the length of time for vocalization play (1 s) and the length of time the fan was powered for odor stimuli (5 s). The offset period was 1 s after the end of the vocalization play and 5 s after the end of the fan-powered odor-stimulus ended. Ongoing activity was taken as the average 2 s baseline firing rate across all stimuli. For computation of membrane potential values, spikes were removed, when present, and the average membrane potential was taken for the entire baseline period (2 s prior to stimulus onset), the time during the stimulus (1 s for vocalizations, 5 s for odors), and the time during the offset period (1 s after vocalization offset, 5 s after odor offset). Electrophysiological data were analyzed blind to the recording location.

Statistical testing was performed in Matlab (MathWorks, Natick, MA) and SigmaPlot (Systat Software Inc., San Jose, CA). For comparisons of categorical variables (behavioral scores), a two-tailed Fisher’s exact test was used. For comparisons of two paired conditions (i.e., baseline vs. odor), data were tested for normality (Shapiro-Wilk). When data were found to be normally distributed, a paired *t*-test (two-tailed) was performed. If data were non-normally distributed, a two-sided Wilcoxon signed rank test was performed. For comparisons between conditions, data were tested for normality (Shapiro-Wilk) and equal variance. When data were found to be normally distributed, an unpaired *t*-test (two-tailed) was performed. When data were non-normally distributed, a two-sided, Mann-Whitney (rank sum) test was performed.

### Reporting summary

Further information on research design is available in the Nature Research Reporting Summary linked to this article.

## Supplementary information

Supplementary Information

Supplementary table

Reporting Summary

## Data Availability

Supporting data are provided. Other materials are available from the corresponding authors upon reasonable request.

## Source data

Source data

## Code availability

Code are available from the corresponding authors upon reasonable request.
