# Supplementary material for: The lateral septum mediates kinship behavior in the rat
Source: Nat Commun. 2020 Jun 22;11:3161. doi: 10.1038/s41467-020-16489-x (PMC7308382; doi:10.1038/s41467-020-16489-x)
Supplement: Supplementary file 1 — Supplementary Information [file 41467_2020_16489_MOESM1_ESM.pdf]

The lateral septum mediates kinship behavior in the rat

Clemens et al.

Supplementary information

Supplementary Figures 1–4

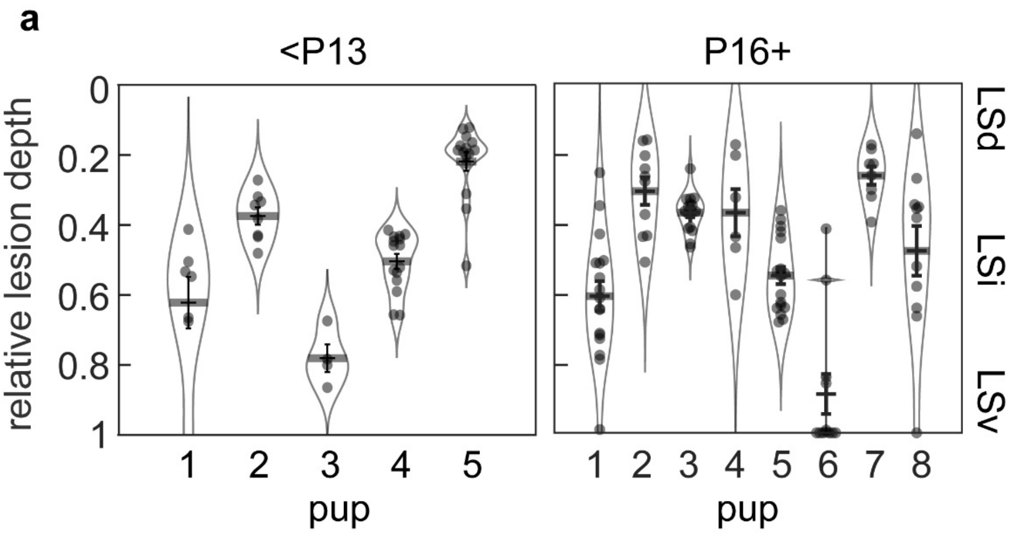

Supplementary Figure 1: Depth analysis of individual pups with lateral septal lesions. a) Mean relative lesion depth for each <P13 lesioned pup (5 pups). Each data point is the relative lesion depth within the lateral septum for each 100 μm section. Horizontal line is the mean depth and error bars are the standard error of the mean (left). Mean relative lesion depth for each P16+ lesioned pup (8 pups). Each data point is the relative lesion depth within the lateral septum for each 100 μm section. Horizontal line is the mean depth and error bars are the standard error of the mean (right). Lateral septum sub-divisions are estimated. Abbreviations: LSd= dorsal lateral septum; LSi= intermediate lateral septum; LSv= ventral lateral septum.

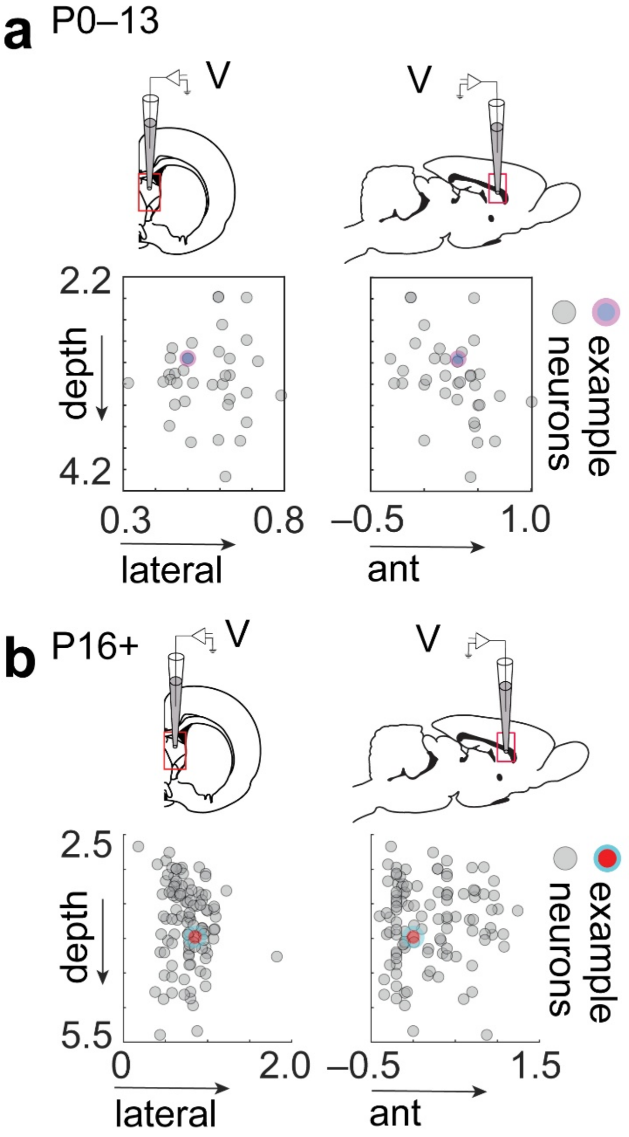

Supplementary Figure 2: a) Estimated locations of cells recorded in juxta-cellular configuration in the P0–13 group. 36 neurons, the example cell is depicted in Figure 4. b) Estimated locations of cells analyzed for supra-threshold responses in the P16+ group. Coordinates for 109 neurons (4 neurons missing coordinate info), the example cell is depicted in figure 5. Coordinates were confirmed from a subset of anatomical recovered cells and adjusted accordingly as specified in the methods section.

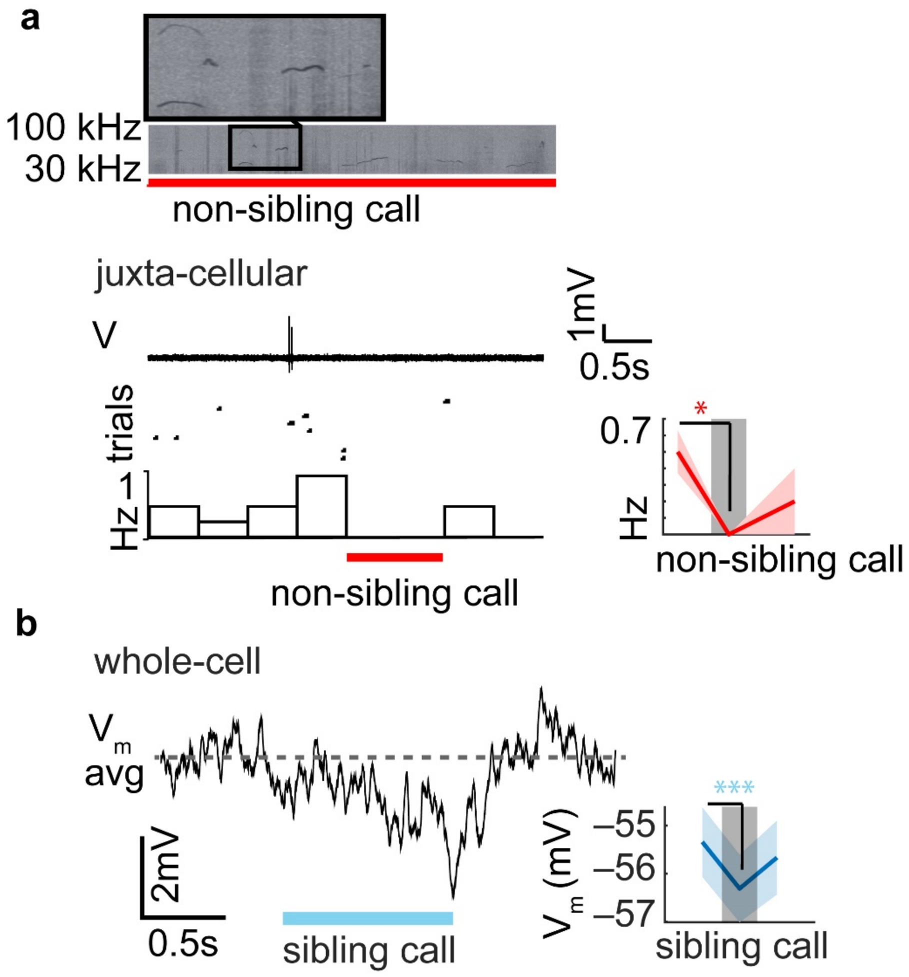

Supplementary Figure 3: Example cells which responded to vocalizations. a) Top: pre-recorded vocalizations from non-sibling rats. 30–100 kHz spectrogram range. Inset: enlargement of vocalizations emitted by non-sibling litter. Bottom: Example trace (top), raster (middle) and peri-stimulus time histogram (bottom) of responses to non-sibling calls (bins 0.5s). Firing rate in baseline period, during call playback and in offset period baseline vs. odor firing rate for non-sibling calls (n=10 trials; baseline median 0.50 Hz, odor median 0.00 Hz, p=0.016, Wilcoxon

signed rank test). b) Example whole-cell patch clamp recorded neuron with significant membrane potential response to calls with hyperpolarization. Blue line represents timing of call playback. Baseline to within sibling call change was significant over trials (n=17 trials; V_m baseline= –55.34 ± 0.72 mV; V_m within call –56.31 ± 0.70 mV; difference: 0.97 ± 0.23 mV; p=0.00053, two-tailed paired t-test). Thick colored line is mean and shaded region is s.e.m. (right a,b). For detailed statistical information, see Supplementary Table 1. *p<0.05, ***p<0.001

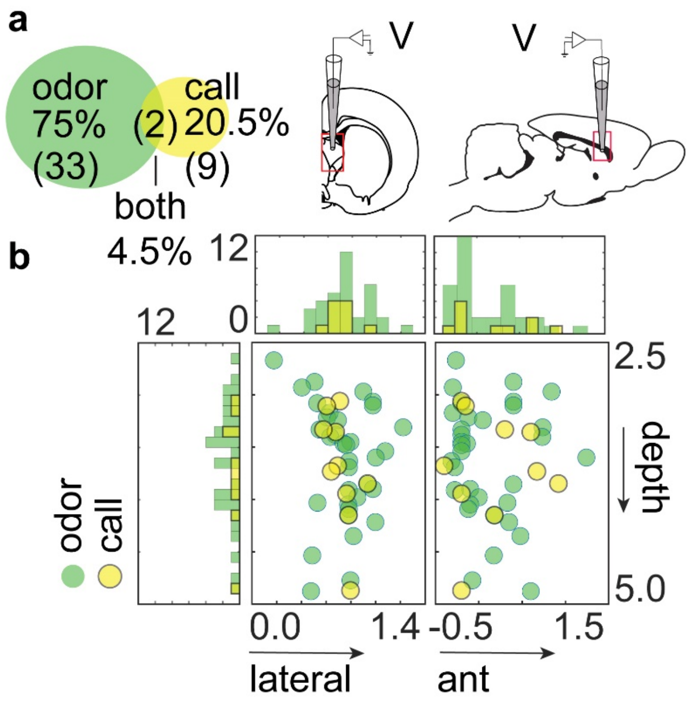

Supplementary Figure 4: Locations of call and odor responsive neurons. a) For P16+ supra-threshold dataset, proportion of significantly responsive neurons responding to odor and call stimuli. b) Locations of significantly responding odor and call responsive neurons are plotted. Coordinates of one call and odor responsive neuron could not be determined and is not included in the map. See methods section for how coordinates were determined based on recovered neuron locations.
