# Supplementary material for: The lateral septum mediates kinship behavior in the rat
Source: Nat Commun. 2020 Jun 22;11:3161. doi: 10.1038/s41467-020-16489-x (PMC7308382; doi:10.1038/s41467-020-16489-x)
Supplement: Supplementary file 2 — Supplementary table [file 41467_2020_16489_MOESM2_ESM.docx]

**Supplementary Table 1: Detailed statistical reporting**

| Figure | n | test | normality/variance | difference | t | power | p-value |
| --- | --- | --- | --- | --- | --- | --- | --- |
| Figure 1d | P0–12: 140 sibling choices, 74 non-sibling choices; P16+: 112 sibling choices, 251 non-sibling choices | Two-tailed Fisher’s exact test | na | na | na | na | ***p = 6.2e-16 |
| Figure 1e | P0–12: 31 pups vs P16+: 41 pups | Unpaired, two-tailed t-test | shapiro-wilk normality test, passed: p=0.806; equal variance test, passed: 0.472 | difference: 0.739; 95 percent confidence interval for difference of means: 0.577 to 0.900 | t=9.117 with 70 degrees of freedom | power with alpha 0.05: 1 | ***p = 1.6e-13 |
| Figure 2d | (<P13 No lesion: 140 sibling choices, 74 non-sibling choices; <P13 lateral septum lesion: 21 sibling choices, 32 non-sibling choices | Two-tailed Fisher’s exact test | na | na | na | na | ***p=  8.9e-4 |
| Figure 2e | <P13 no lesion (31 pups) vs. <P13 LSL (5 pups) | Unpaired, two-tailed t-test | shapiro-wilk normality test, passed: p=0.721; equal variance test, passed: p=0.359 | difference: 0.581; 95 percent confidence interval for difference of means: 0.183 to 0.978 | t = 2.970 with 34 degrees of freedom | Power of performed test with alpha = 0.050: 0.788 | **p = 0.005 |
| In text comparison | <P13 LS lesion scores(21 sibling choice, 32 non-sibling choices) vs. 50/50 performance(27 sibling vs 27 non-sibling choices) | Two-tailed Fisher’s exact test | na | na | na | na | p = 0.333 |
| In text comparison | P16+ LS lesion scores (24 sibling choices, 24 non-sibling choices) vs. 50/50 performance (24 sibling choices, 24 non-sibling choices) | Two-tailed Fisher’s exact test | na | na | na | na | p = 1 |
| Figure 3d  No lesion vs. LS lesion | P16+ no lesion: 112 sibling choices, 251 non-sibling choices; P16+ lateral septal lesion: 24 sibling choices, 24 non-sibling choices; | Two-tailed Fisher’s exact test | na | na | na | na | *p = 0.014 |
| Figure 3d  No lesion vs. P16+ ctx lesion | P16+ no lesion: 112 sibling choices, 251 non-sibling choices; P16+ ctx lesion: 13 sibling choices, 37 non-sibling choices; | Two-tailed Fisher’s exact test | na | na | na | na | p = 0.517 |
| Figure 3d  P16+ ctx lesion vs. P16+ LS lesion | P16+ ctx lesion: 13 sibling choices, 37 non-sibling choices;  P16+ LS lesion: 24 sibling choices, 24 non-sibling choices; | Two-tailed Fisher’s exact test | na | na | na | na | *p = 0.021 |
| Figure 3e  No lesion vs. LS lesion | No lesion, n=41; LS lesion, n=12 | Unpaired, two-tailed t-test | shapiro-wilk normality test, passed: p=0.753, equal variance test, passed: 0.102 | difference: -0.317, 95 percent confidence interval for difference of means: -0.561 to -0.0726 | t = -2.605 with 51 degrees of freedom | power with alpha 0.05 = 0.657 | *p = 0.012 |
| Figure 3e  No lesion vs. P16+ ctx lesion | No lesion, n=41; LS lesion, n=9 | Unpaired, two-tailed t-test | Normality Test (Shapiro-Wilk) Passed (P = 0.308)  Equal Variance Test: Passed (P = 0.662) | Difference 0.0149; 95 percent confidence interval for difference of means: -0.206 to 0.235 | t=0.136 with 48 degrees of freedom | Power of performed test with alpha = 0.050: 0.050 | p = 0.893 |
| Figure 3e  P16+ ctx lesion vs. P16+ LS lesion | LS lesion, n=12; Ctx lesion, n=9 | Unpaired, two-tailed t-test | shapiro-wilk normality test, passed: p=0.085; equal variance test, passed: 0.396 | difference: 0.332; 95 percent confidence interval for difference of means: -0.0731 to 0.736 | t = 1.715 with 19 degrees of freedom | power with alpha 0.05 = 0.249 | p=0.103 |
| Figure 4c | sibling odor  n=15 trials | Paired, two-tailed, t-test | shapiro-wilk normality test, passed: p=0.112 | baseline mean ± SEM = 0.133 ± 0.103 Hz, odor mean ± SEM = 1.093 ± 0.223 Hz | t=4.618 with 14 degrees of freedom | power with alpha 0.05 = 0.992 | ***p = 4.0e-4 |
|  | Non-sibling odor,  n=16 trials | Paired, two-tailed, t-test | shapiro-wilk normality test, passed: p=0.811 | baseline mean ± SEM = 0.594 ± 0.267 Hz, odor mean ± SEM = 0.725 ± 0.211 Hz, difference mean ± SEM = 0.131 ± 0.340, | t=0.386 with 15 degrees of freedom | power with alpha 0.05 = 0.050 | p = 0.705 |
| Figure 4e | mom odor, n=15 trials | Wilcoxon signed rank test | shapiro-wilk normality test, failed, p<0.05 | baseline median = 0.00 Hz, odor median = 2.00 Hz | z-statistic based on positive ranks = 3.177 |  | ***p = 3.7e-4 |
|  | Non-mom odor, n=16 trials | Paired, two-tailed, t-test | shapiro-wilk normality test, passed: p=0.334 | baseline mean ± s.e.m. = 0.25 ± 0.11 Hz; odor mean ± s.e.m. = 1.40 ± 0.24 Hz, difference mean ± s.e.m. = 1.15 ± 0.19 | t=6.047 with 15 degrees of freedom | power with alpha 0.05 = 1 | ***p = 2.2e-5 |
| Figure 5c | Sibling odor, n=8 trials | Paired, two-tailed, t-test | shapiro-wilk normality test, passed: p=0.284 | baseline mean ± SEM = 5.125 ± 1.636 Hz, odor mean ± SEM = 13.375 ± 2.796 Hz, difference mean ± SEM = 8.250 ± 3.222 | t=2.561 with 7 degrees of freedom | power with alpha 0.05 = 0.525 | *p = 0.038 |
|  | Non-sibling odor (n=8 trials | Paired, two-tailed, t-test | shapiro-wilk normality test, passed: p=0.164 | baseline mean ± s.e.m. = 5.313 ± 1.285 Hz, odor mean ± SEM = 12.625 ± 1.037 Hz, difference mean ± SEM = 7.313 ± 1.549 | t=4.720 with 7 degrees of freedom | power with alpha 0.05 = 0.980 | **p = 0.002 |
| Figure 5e | Mom odor, n=8 trials | Paired, two-tailed, t-test | shapiro-wilk normality test, passed: p=0.749 | baseline mean ± s.e.m. = 10.375 ± 2.289 Hz, odor mean ± s.e.m. = 9.475 ± 1.381 Hz, difference mean ± s.e.m. = 0.900 ± 1.426 | t=0.631 with 7 degrees of freedom | power with alpha 0.05 = 0.05 | p = 0.548 |
|  | Non-mom odor, n=8 trials | Paired, two-tailed, t-test | shapiro-wilk normality test, passed: p=0.658 | baseline mean ± SEM = 8.250 ± 2.246 Hz, odor mean ± SEM = 8.875 ± 2.074 Hz, difference mean ± SEM = 0.625 ± 1.760 | t=0.355 with 7 degrees of freedom | power with alpha 0.05 = 0.05 | p = 0.733 |
| Figure 6c | sibling odor firing rates, n=16 trials | Paired, two-tailed, t-test | shapiro-wilk normality test, passed: p=0.297 | baseline mean ± SEM = 0.938 ± 0.254 Hz, odor mean ± SEM = 2.337 ± 0.350 Hz, difference mean ± SEM = 1.400 ± 0.437 | t=3.201 with 15 degrees of freedom | power with alpha 0.05 = 0.824 | **p = 0.006 |
|  | non-sibling odor firing rates, n=16 trials | Paired, two-tailed, t-test | shapiro-wilk normality test, passed: p=0.996 | baseline mean ± s.e.m. = 0.500 ± 0.129 Hz, odor mean ± s.e.m. = 3.663 ± 0.513 Hz, difference mean ± s.e.m. = 3.163 ± 0.469 | t=6.739 with 15 degrees of freedom | power with alpha 0.05 = 1 | ***p = 6.7e-6 |
|  | sibling vs. non-sibling odor firing rates, n=16 trials | Paired, two-tailed, t-test | shapiro-wilk normality test, passed: p=0.129 | baseline mean ± SEM = 2.337 ± 0.350 Hz, odor mean ± SEM = 3.663 ± 0.513 Hz, difference mean ± SEM = 1.325 ± 0.426 | t=3.112 with 15 degrees of freedom | power with alpha 0.05 = 0.798, | **p = 0.007 |
|  | sibling odor V_m_, n=16 trials | Paired, two-tailed, t-test | shapiro-wilk normality test, passed: p=0.395 | baseline mean ± SEM = –62.063 ± 0.786 mV, odor mean ± SEM = –62.591 ± 0.659 mV, difference mean ± SEM = 0.528 ± 0.205 | t=2.573 with 15 degrees of freedom | power with alpha 0.05 = 0.60 | *p = 0.021 |
|  | non-sibling odors, n=16 presentations | Paired, two-tailed, t-test | shapiro-wilk normality test, passed: p=0.967 | baseline mean ± s.e.m.= –62.028 ± 0.582 mV, odor mean ± s.e.m.= –62.601 ± 0.599 mV, difference mean ± s.e.m.= 0.573 ± 0.182 | t=3.148 with 15 degrees of freedom | power with alpha 0.05 = 0.809 | **p = 0.007 |
| In text correlation | Correlation of ongoing firing rate vs. age (n=149 neurons) | Spearman Rank Order Correlation |  | Correlation Coefficient: 0.316 |  | power with alpha 0.05 = 0.977 | p = 8.6e-5 |
| Figure 7a | P0–13 (n=36); P16+ (n=113); ongoing firing rates | Mann-Whitney Rank Sum Test | Normality Test (Shapiro-Wilk) Failed (P < 0.050) | P0–13 median=0.59, mean ± s.e.m.=1.59 ±0.73  P16+ median=1.12,  mean ± s.e.m.= 2.46±0.29 | Mann-Whitney U Statistic= 1376.000  T = 2042.000 |  | **p=  0.004 |
| Figure 7d | P0–13 (sibling odor responsive, n=8, non-significant, n=26) P16+ (sibling odor responsive, n=9, non-significant, n=100) | Two-tailed Fisher’s exact test | na | na | na | na | *p = 0.029 |
|  | P0–13 (non-sibling odor responsive, n=5, non-significant, n=30) P16+ (non-sibling odor responsive, n=13, non-significant, n=97) | Two-tailed Fisher’s exact test | na | na | na | na | P = 0.77 |
| Figure 8b | non-sibling odor, n=5 trials | Paired, two-tailed, t-test | Normality Test (Shapiro-Wilk), Passed, p = 0.155 | baseline mean ± s.e.m.= 6.40 ± 0.678 Hz, odor mean 8.92 ± 0.625 Hz, difference = 2.52 Hz; 95 percent confidence interval for difference of means: 3.611 to 1.429 | t = –6.413 with 4 degrees of freedom | power of test with alpha 0.05: 0.996 | **p= 0.003 |
| Figure 8c | Sibling odor, n=5 trials | Paired, two-tailed, t-test | Normality Test (Shapiro-Wilk), passed, p= 0.686 | baseline mean ± s.e.m.= 1.90 ± 0.187 Hz; odor mean 1.04 ± 0.075 Hz, difference = 0.86 Hz; 95 percent confidence interval for difference of means: 0.188 to 1.532 | t = 3.553 with 4 degrees of freedom | power of test with alpha 0.05: 0.728 | *p = 0.024 |
|  | mother odor ,n=5 trials | Paired, two-tailed, t-test | Normality Test (Shapiro-Wilk), Passed, p = 0.391 | baseline mean 1.60 ± 0.245 Hz, odor mean 0.680 ± 0.080 Hz, difference = 0.920 Hz; 95 percent confidence interval for difference of means: 0.183 to 1.657 | t = 3.467 with 4 degrees of freedom | power of test with alpha 0.05: 0.706 | *p = 0.026 |
| Figure 8d | mother odor, n=6 trials | Paired, two-tailed, t-test | Normality Test (Shapiro-Wilk), Passed, p = 0.942 | baseline mean 3.00 ± 0.764 Hz, odor mean 8.57 ± 1.475 Hz, difference = 5.57 Hz, 95 percent confidence interval for difference of means: 9.736 to 1.397 | t = 3.432 with 5 degrees of freedom | power of test with alpha 0.05: 0.752 | *p = 0.019 |
| Figure 8e | Sibling vs. non-sibling depth comparison, sibling odor neurons, n=8; non-sibling odor neurons, n=12 | unpaired, two-tailed, t-test | shapiro-wilk normality test, passed: p=0.082, equal variance test, passed: p = 0.884 | difference: 627.125 µm, 95 percent confidence interval for difference of means: 237.304 to 1016.946 | t = 3.380 with 18 degrees of freedom | power with alpha 0.05 = 0.878 | **p = 0.003 |
|  | Mother vs. non-mother depth comparison, mother odor neurons, n=8; non-mother odor neurons, n=7 | unpaired, two-tailed, t-test | shapiro-wilk normality test, passed: p=0.293, equal variance test, passed: 0.961 | difference= 688.036 µm; 95 percent confidence interval for difference of means: 75.367 to 1300.705 | , t = 2.426 with 13 degrees of freedom | power with alpha 0.05 = 0.529 | *p = 0.031 |
| Supplementary Figure 3b | non-sibling calls, n=10 trials | Wilcoxon signed rank test | shapiro-wilk normality test, failed, p<0.050 | baseline median 0.500 Hz, odor median 0.000 Hz | z-statistic based on positive ranks = -2.428 |  | *p=0.016 |
|  | Sibling calls, n=17 trials | Paired, two-tailed, t-test | Normality Test (Shapiro-Wilk), Passed, p=0.792) | V_m_ baseline= –55.342 ± 0.723 mV; V_m_ within call –56.312 ± 0.702 mV | t=4.318 with 16 degrees of freedom | power of test with alpha 0.05: 0.984 | ***p = 0.00053 |
